# Comparison of therapeutic strategies in patients presenting with left atrial thrombus despite oral anticoagulation

**DOI:** 10.1007/s00392-026-02917-3

**Published:** 2026-04-20

**Authors:** Tobias Schreiber, Patrick Nagel, Johannes Lucas, Anja Cretnik, Laura Isabel Adler, Verena Tscholl, Ulf Landmesser, Gerhard Hindricks, Anna Sannino, Andi Rroku, Martin Huemer, Philipp Attanasio

**Affiliations:** 1 Deutsches Herzzentrum der Charité, Klinik für Kardiologie, Angiologie und Intensivmedizin, Charité - Universitätsmedizin Berlin, Campus Benjamin Franklin, Hindenburgdamm 30, 12203 Berlin, Germany; 2 German Centre for Cardiovascular Research (DZHK), Berlin, Germany; 3 Berlin Institute of Health (BIH), Berlin, Germany; 4 Friede Springer - Cardiovascular Prevention Center @Charité Universitaetsmedizin Berlin, Hindenburgdamm 30, 12203 Berlin, Germany

**Keywords:** Atrial fibrillation, Left atrial appendage thrombus, Oral anticoagulation, Thrombus resolution

## Abstract

**Background:**

Left atrial appendage (LAA) thrombus formation is associated with elevated stroke risk and mortality. This study was designed to compare different therapeutic strategies in patients presenting with LAA thrombi despite adequate oral anticoagulation (OAC) therapy.

**Methods:**

In this retrospective single-center study, patients with atrial fibrillation (AF) and LAA thrombus despite adequate OAC for more than three weeks were identified. A follow-up transesophageal echo (TEE) was performed at least four weeks after the initial TEE. Thrombus resolution was assessed for each treatment cycle, defined as the interval of OAC therapy between two consecutive TEE examinations.

**Results:**

The study included 216 patients who underwent a total of 294 treatment cycles. At baseline, 47% (*n* = 101) of patients were receiving novel oral anticoagulants (NOACs), while 53% (*n* = 115) were treated with vitamin-K antagonists (VKAs). Treatment options included switching OAC from VKA to NOAC (*n* = 18), from NOAC to a different NOAC (*n* = 14) and from NOAC to VKA (*n* = 77); or maintaining the same NOAC (*n* = 28) or VKA (*n* = 157). Overall, LAA thrombi resolved in 70% (152/216) after a mean follow up time of 130 (SD 195) days). No significant differences regarding resolution rate between the five different anticoagulation strategies were observed (*p* = 0.866). Multivariate regression analysis identified tricuspid annular plane systolic excursion as independently predictive of LAA thrombus persistence (OR 0.87; 95% CI 0.78–0.98; *p* = 0.026).

**Conclusion:**

This is the largest cohort of patients presenting with LAA thrombi despite adequate OAC. Overall resolution was 70%. Modification of the anticoagulation regimen did not result in higher thrombus resolution rates compared with continuation of the same therapy.

**Graphical Abstract:**

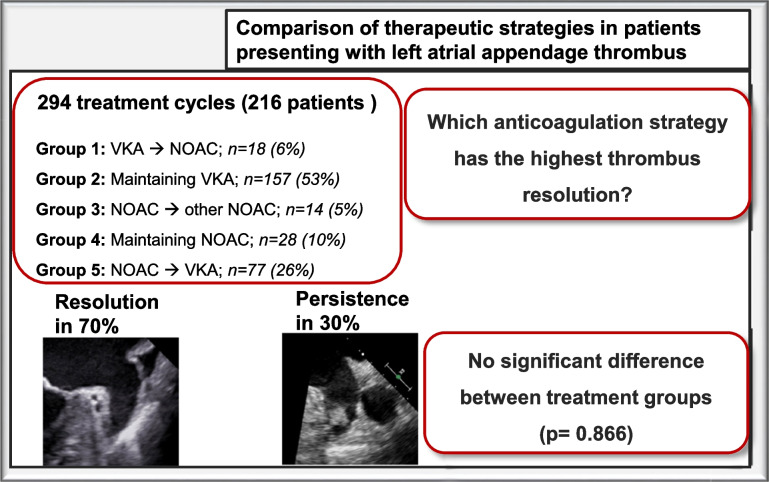

## Background

Despite guideline-directed oral anticoagulation (OAC) therapy, left atrial appendage (LAA) thrombus formation occurs in patients with atrial fibrillation (AF) with a prevalence of up to 5% [1]. Risk factors include patients with a higher CHA_2_DS_2_-Vasc score, obesity, inadequately reduced OAC dosages and concomitant medication affecting therapeutic efficacy [2, 3]. The presence of LAA thrombus is clinically relevant, as it is associated with increased mortality and a higher risk of systemic thromboembolism, and delays rhythm control therapy [4].

Management of patients presenting with LAA thrombus despite OAC is unclear. In clinical practice, approaches include switching to another novel oral anticoagulant (NOAC), switching from NOAC to vitamin-K antagonists (VKA) and vice versa, or to remain on the same treatment [5]. While data suggests that in OAC-naïve patients, NOAC are associated with a higher probability of LAA thrombus resolution [2, 6, 7], findings in patients with persistent LAA thrombus are more inconsistent: a recently published study [8] reported higher resolution rates for VKA (OR 3.23; CI 1.03–10.1, *p* = 0.04), whereas other studies [9, 10] found no evidence of superiority. This study sought to retrospectively compare these treatment options in a larger cohort, regarding efficacy and safety, to help guide treatment decisions for this patient group. Emphasis was put on echocardiographic properties to explore possible factors predicting thrombus resolution.

## Methods

This study represents a retrospective chart review of patients presenting for rhythm control therapy at Deutsches Herzzentrum of Charité – Universitätsmedizin Berlin between 2010 and 2023. The search criteria within the hospital’s patient information system were performed using International Classification of Diseases (ICD) and Operation and Procedure (OPS) codes and included the presence of AF, the availability of at least two transesophageal echoes (TEE), and the presence of an intracardiac thrombus. Medical history, comedication, echocardiographic and laboratory results were retrieved from the medical records. Duration of follow-up and factors that potentially influence thrombus resolution were recorded.

### Inclusion and exclusion criteria

Inclusion criteria were the presence of a LAA thrombus in patients with non-valvular AF diagnosed by TEE despite adequate OAC intake for more than three weeks and the availability of a follow-up TEE performed at least four weeks after the initial TEE [11]. Exclusion criteria were moderate to severe mitral valve stenosis, and patients who had undergone LAA closure (surgical or device).

### Endpoints

Primary endpoint was thrombus resolution enabling rhythm control therapy, as assessed in TEE. In some patients, more than one TEE for follow-up was available; therefore, multiple treatment cycles could be analyzed in case of thrombus persistence. One treatment cycle was defined as treatment with OAC between two TEE examinations. To compare clinical and echocardiographic characteristics, patients were divided into responders (resolution at one of the follow-up TEEs) and non-responders (no resolution at all). Secondary endpoints were adverse events related to OAC, including stroke and major bleeding, during follow-up. Major bleeding was defined according to the Bleeding Academic Research Consortium (BARC) classification [12].

### Transesophageal echocardiography

TEE was selected as the sole imaging modality, as it remains the gold standard for detection of LAA thrombi [13]. TEE were performed with commercially available Vivid e95 cardiovascular ultrasound systems (GE Healthcare, Horten, Norway). During TEE, the LAA was visualized in multiple and standardized views, according to current recommendations [14]. Thrombi of the LAA were defined as echodense masses that are distinct from pectinate muscles and the underlying endocardium [15]. The differentiation between LAA thrombus towards LAA sludge and spontaneous echo contrasts (SEC) was performed according to EACVI/EHRA recommendations [13]. Briefly, sludge was defined as dense smoke with not solid, viscid echodensity; SEC was defined as low swirling patterns with intense echodensity. Cases where differentiation between thrombus and sludge/severe SEC was not unequivocally possible were rated as grade 1 LAA thrombus. Grade 2 thrombus was defined as solid thrombus, Grade 3 thrombus was defined as thrombus occupying more than 50% of the LAA (see Picture 1) [9, 16]. LAA morphology was classified into windsock, chicken-wing and cactus/cauliflower morphology [17, 18]. LAA ostium size was measured from the level of the left circumflex artery towards a point 10 mm lower than the top of the ridge. Ostium size and depth were measured twice for each patient (see Picture 2) using a biplane, perpendicular view [16]. For statistical analysis, mean values were used.

**Picture 1:**
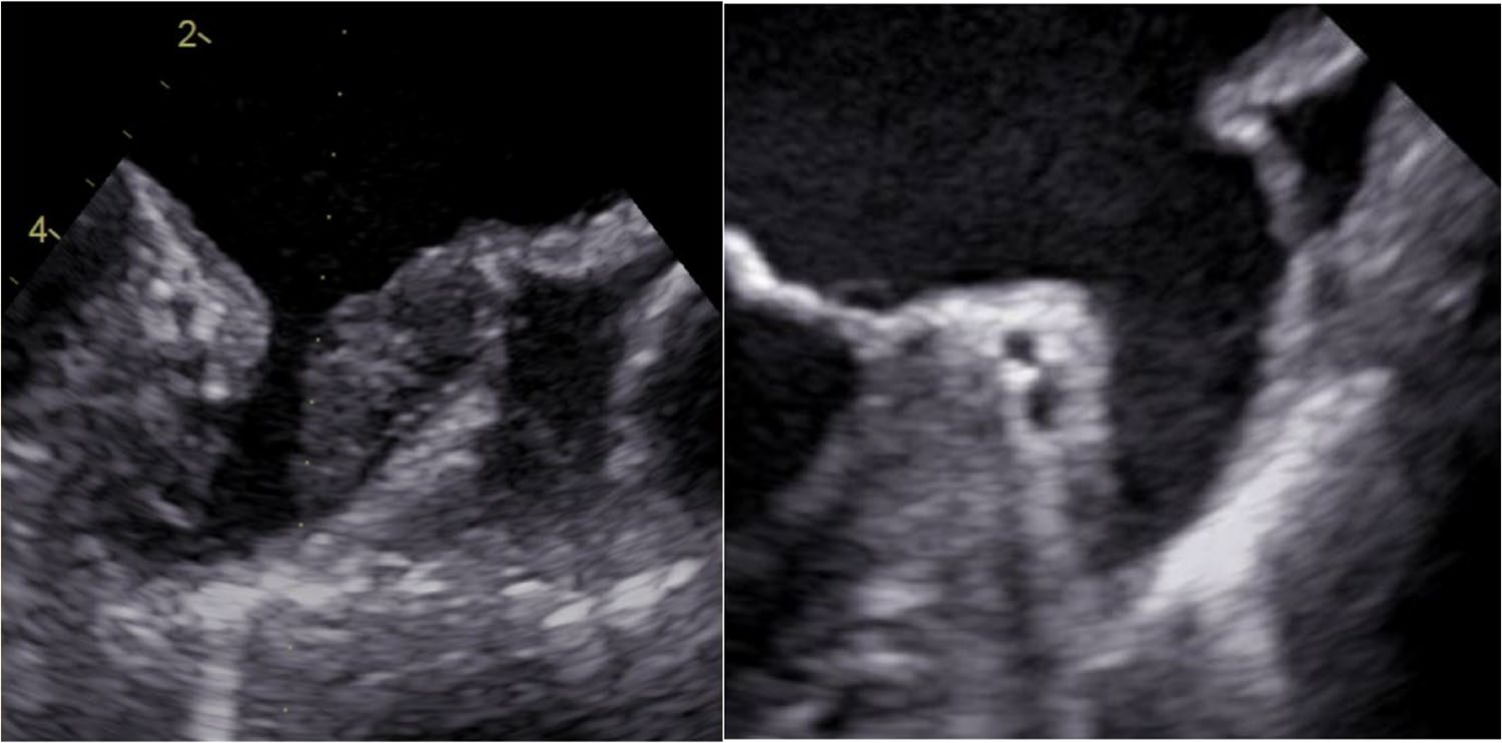
Identification of a solid thrombus occupying >50% of the LAA (“grade 3”). Transesophageal echocardiography eight weeks after showed thrombus resolution

**Picture 2:**
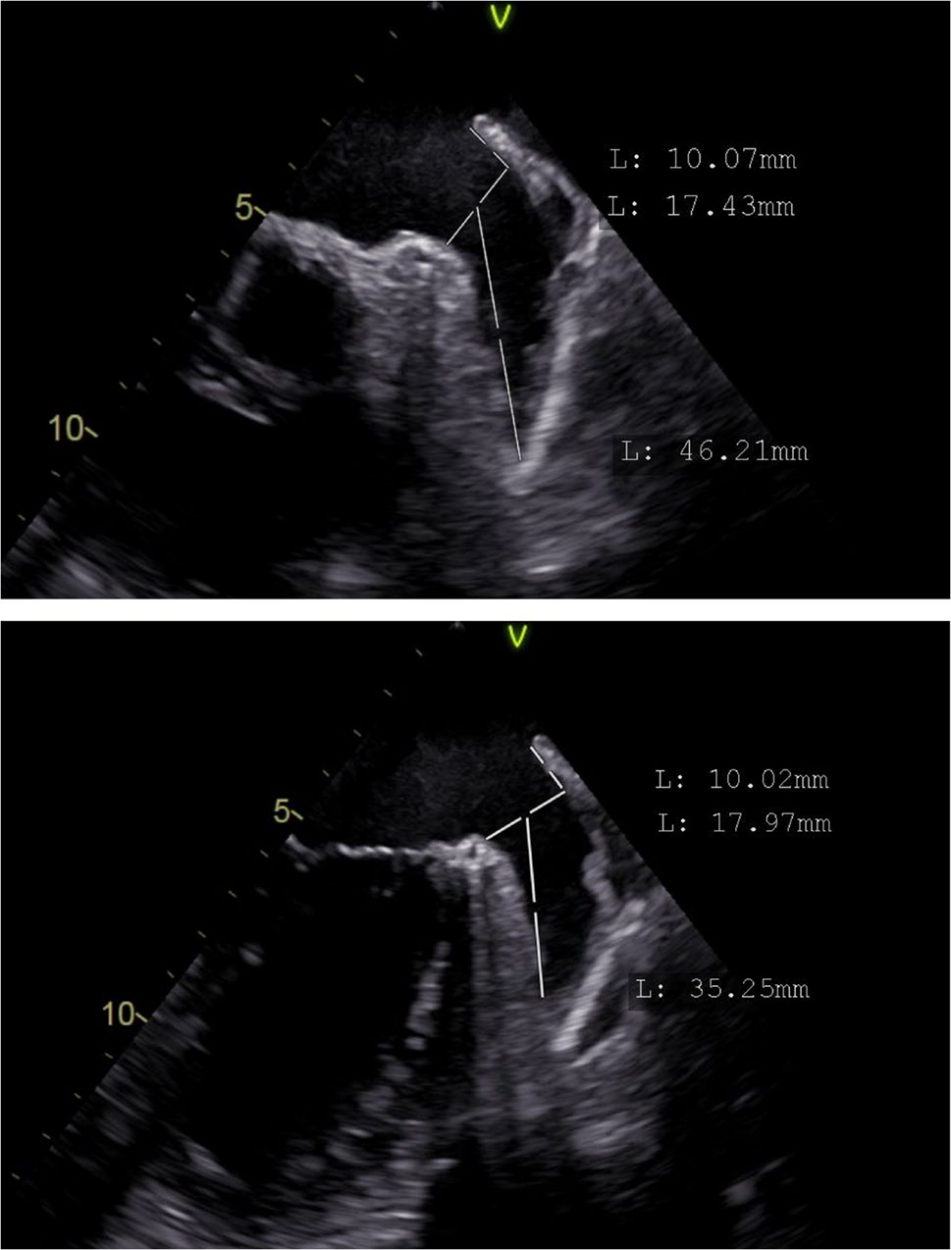
Measurement of left atrial appendage ostium and length using a biplane view (45 and 90 degrees)

Treatment strategies were chosen according to patients’ characteristics as well as the physician’s and patients’ preference. The study was approved by the local Ethics Committee of Charité – Universitätsmedizin Berlin (EA4/122/21).

### Transthoracic echocardiography

Standard echocardiographic parameters, including left ventricle dimensions and function, were obtained according to recommendations from the American Society of Echocardiography [19]. Left atrial strain was measured in a single apical four-chamber view using TomTec software (TomTec, Unterschleissheim, Germany). Since a relevant number of patients were expected to have AF, only reservoir strain was considered in this analysis, excluding conduit and contraction strain [20]. Right ventricular systolic function was assessed using tricuspid annular plane systolic excursion (TAPSE), measured in the apical four-chamber view with M-mode at the lateral tricuspid annulus [21]. Tricuspid regurgitation severity was evaluated using an integrated multiparametric approach. TR was graded using a three-grade classification system (mild, moderate, severe). In this framework, the severe TR category encompassed all advanced forms of regurgitation, including what is now referred to as massive and torrential TR, as the expanded five-grade grading scheme had not yet been introduced at the time of data acquisition. For the purpose of this analysis, moderate and severe were considered as significant and analyzed as one group. Aortic and mitral regurgitation were assessed according to current guidelines recommendations [22].

### Statistical analysis

Data are presented as absolute numbers, as percentages for categorical variables or mean ± standard deviation (SD) for continuous variables. Categorical variables were compared using the *χ*^2^ test. The independent sample *t*-test (for normally distributed data) or the Mann–Whitney *U* test (for skewed data) was used to compare continuous variables. For normally distributed data, the relationship between left atrial thrombus persistence and potential influencing factors was calculated with Pearson’s correlation coefficient and Spearman for skewed data. Multivariate logistic regression analysis was used to assess the strength of the relationship between thrombus resolution and potential influencing factors based on univariate analysis and clinically relevant factors. Results were reported as odds ratios (ORs) with corresponding 95% confidence intervals (CI). Analyses were performed using SPSS software version 22 (SPSS Inc., Chicago, IL, USA). We used a two-sided significance level of <0.05.

## Results

Our database query identified 216 patients, which were included for further analysis. Median age was 70.99 years (IQR 12), 89 patients (41%) were female. All patients had OAC at baseline, in appropriate dosage according to age, weight and renal function. All patients were scheduled for TEE before cardioversion or catheter ablation. At baseline, 47% (*n* = 101) of patients were receiving NOAC, while 53% (*n* = 115) were treated with VKA. Patient characteristics are shown in Table 1.

**Table 1:** Patient characteristics

Parameter	All (*n* = 216)	Responders (*n* = 152)	Non-responders (*n* = 64)	*p*-value
Female gender, *n* (%)	89 (41)	61 (40)	28 (44)	0.652
Age, median (years; IQR)	72 (13)	72 (13)	75 (14)	0.095
BMI, median (kg/m^2^; IQR)	27.5 (6.0)	27.5 (6.4)	27.3 (5.0)	0.672
Paroxysmal AF, *n* (%)	55 (25)	38 (25)	17 (27)	0.792
Coronary heart disease, *n* (%)	96 (44)	64 (42)	32 (50)	0.298
Dilatative cardiomyopathy, *n* (%)	17 (8)	11 (7)	6 (9)	0.590
CHA_2_DS_2_-Vasc, median (IQR)	4.0 (2.0)	4.0 (2.0)	5.0 (1.0)	**0.042**
Arterial hypertension, *n* (%)	163 (75)	116 (76)	47 (73)	0.729
Prior TIA/stroke, *n* (%)	31 (14)	19 (13)	12 (19)	0.288
Prior myocardial infarction, *n* (%)	30 (14)	20 (13)	10 (16)	0.669
Prior vein thrombosis, *n* (%)	9 (4)	6 (4)	3 (5)	0.726
Prior pulmonary embolism, *n* (%)	8 (4)	5 (3)	3 (5)	0.697
Mechanical valve, *n* (%)	3 (1)	3 (2)	0 (0)	0.556
Diabetes mellitus type II, *n* (%)	64 (30)	43 (28)	21 (33)	0.518
Chronic obstructive pulmonary disease, *n* (%)	30 (14)	18 (12)	12 (19)	0.199
Intake of VKA at baseline, *n* (%)	115 (53)	78 (51)	37 (58)	0.456
Intake of NOAC at baseline, *n* (%)	101 (47)	74 (49)	27 (42)	0.456
Intake of rivaroxaban at baseline, *n* (%)	31 (14)	22 (14)	9 (14)	1.00
Intake of edoxaban at baseline, *n* (%)	13 (6)	10 (7)	3 (5)	0.759
Intake of apixaban at baseline, *n* (%)	52 (24)	38 (25)	14 (22)	0.728
Intake of dabigatran at baseline, *n* (%)	5 (2)	4 (3)	1 (2)	1.00
Switch to any other OAC, *n* (%)	103 (48)	70 (46)	33 (52)	0.277
Concomitant intake of ASS, *n* (%)	48 (22)	29 (19)	19 (30)	0.105
Concomitant intake of clopidogrel, *n* (%)	26 (12)	16 (11)	10 (16)	0.360
Amiodarone, *n* (%)	13 (6)	5 (3)	8 (13)	**0.023**
Betablocker, *n* (%)	202 (94)	142 (93)	60 (94)	1.00
Digitalis, *n* (%)	64 (30)	49 (32)	15 (23)	0.197
Major bleeding during follow-up, *n* (%)	6 (3)	4 (3)	2 (3)	0.662
Stroke during follow-up, *n* (%)	1 (0.5)	1 (0.7)	0 (0)	0.534

*AF* atrial fibrillation, *BMI* body mass index, *NOAC* novel oral anticoagulation, *OAC* oral anticoagulation, *TIA* transient ischemic attack, *VKA* vitamin-K antagonist

### Treatment cycles and resolution rate

The 216 patients underwent a total of 294 treatment cycles with a mean treatment cycle number of 1.36 (SD 0.71). 159 (74%) patients had one treatment cycle, 43 (20%) patients had two treatment cycles, and 14 (6%) patients had three or more treatment cycles.

Five treatment strategies were identified:

- Group 1 (Changing from VKA to NOAC) – *n* = 18 (6%)
- Group 2 (Maintaining VKA therapy with enhanced time in the therapeutic range) – *n* = 157 (53%)
- Group 3 (Changing from NOAC to another NOAC) – *n* = 14 (5%)
- Group 4 (Maintaining the same NOAC therapy) – *n* = 28 (10%); and
- Group 5 (Changing from NOAC to VKA) – *n* = 77 (26%).

After a mean follow up time of 130 (SD 195) days, LAA thrombi resolved in 70% (152/216; responders); in the rest of the cases, no thrombus resolution was observed (non-responders). Detailed information comparing patients with and without thrombus resolution is given in Tables 2 and 3.

**Table 2:** Laboratory and transthoracic echocardiographic markers at baseline

Parameter	All (*n* = 216)	Responders (*n* = 152)	Non-responders (*n* = 64)	*p*-value
Hemoglobin, median (mg/dl; IQR)	13.80 (2.60)	13.80 (2.55)	13.60 (2.60)	0.910
Creatinine level, median (mg/dl; IQR)	1.06 (0.47)	1.05 (0.38)	1.11 (0.74)	0.522
eGFR, median (ml/min; IQR)	62.48 (30.72)	62.78 (28.24)	60.90 (30.08)	0.144
Thrombocyte count, median (n/l; IQR)	209.00 (89.00)	214.00 (75.00)	194.50 (104.00)	0.126
NT-pro BNP, median (ng/l; IQR)	2907 (4732)	2832 (4684)	3569 (10290)	0.203
INR before TEE 1, median (IQR)	1.39 (0.72)	1.38 (0.73)	1.40 (0.64)	0.535
LVEF, median (%, IQR)	50.00 (18)	50.00 (18)	50.00 (20)	0.675
Moderate to severe aortic valve insufficiency, *n* (%)	12 (6)	8 (5)	4 (6)	0.407
Moderate to severe aortic valve stenosis, *n* (%)	11 (5)	7 (5)	4 (6)	0.335
Moderate to severe mitral valve insufficiency, *n* (%)	75 (35)	52 (34)	23 (36)	0.476
Moderate to severe tricuspid valve insufficiency, *n* (%)	66 (31)	42 (28)	24 (38)	**0.024**
Interventricular septum diameter, median (mm; IQR)	12.00 (3.00)	12.00 (3.00)	12.00 (3.00)	0.163
Left ventricular end-diastolic diameter, median (mm; IQR)	49.00 (11.25)	50.00 (11.25)	48.50 (10.75)	0.105
Left atrial volume index, mean (ml/m^2^; SD)	48.67 (18.13)	48.30 (19.34)	49.66 (14.48)	0.472
Left atrial reservoir strain, median (%; IQR)	9.40 (8.00)	9.60 (8.20)	9.25 (7.13)	0.342
E/A ratio, mean (SD)	2.46 (1.39)	2.45 (1.44)	2.57 (1.01)	0.760
E-wave, median (m/s; IQR)	0.96 (0.41)	0.95 (0.35)	1.00 (0.50)	0.348
A-wave, median (m/s; IQR)	0.40 (0.27)	0.40 (0.31)	0.33 (0.25)	0.407
Tricuspid annular plane systolic excursion, mean (mm; SD)	17.47 ± 4.78	18.33 ± 4.52	14.94 ± 4.68	**<0.001**

*eGFR* estimated glomerular filtration rate, *LVEF* left ventricular ejection fraction, *TEE* transesophageal echocardiography

**Table 3:** Thrombus and left atrial appendage characteristics

Parameter	All (*n* = 216)	Responders (*n* = 152)	Non-Responders (*n* = 64)	*p*-value
Left atrial appendage flow rate, median (m/s; IQR)	0.20 (0.15)	0.20 (0.15)	0.16 (0.10)	0.018
LAA ostium diameter, median (mm; IQR)	18.5 (5.0)	18.5 (5.0)	19.0 (3.75)	0.286
LAA length, median (mm; IQR)	29.5 (8.0)	29.25 (8.13)	29.5 (9.75)	0.975
Chicken-wing morphology, *n* (%)	19 (9)	11 (7)	8 (13)	0.106
Cactus/cauliflower morphology, *n* (%)	90 (42)	63 (41)	27 (42)	0.257
Windsock morphology, *n* (%)	63 (37)	51 (41)	12 (26)	0.076
Morphology not specifiable, *n* (%)	44 (20)	27 (18)	17 (27)	0.101
Thrombus located at apex, *n* (%)	91 (42)	67 (44)	24 (38)	0.421
Thrombus located at ostium, *n* (%)	17 (8)	15 (10)	2 (3)	0.101
Thrombus located at body, *n* (%)	59 (27)	39 (26)	20 (31)	0.120
Mobile thrombus, *n* (%)	23 (11)	15 (10)	8 (13)	0.247
Grade I (soft) thrombus, *n* (%)	83 (38)	59 (39)	24 (38)	0.491
Grade II thrombus (<50% of the LAA), *n* (%)	130 (60)	91 (60)	39 (61)	0.504
Grade III thrombus (>50% of the LAA), *n* (%)	3 (1)	2 (1)	1 (2)	0.654
Thrombus width, median (mm; IQR)	9.50 (8.3)	10.00 (9.0)	9.00 (4.3)	0.576
Thrombus length, median (mm; IQR)	8.00 (4.0)	8.00 (4.0)	7.00 (5.5)	0.973
Sinus rhythm at TEE1, *n* (%)	10 (5)	9 (6)	1 (2)	0.258

*LAA* left atrial appendage, *TEE* transesophageal echocardiography

**Fig. 1:**
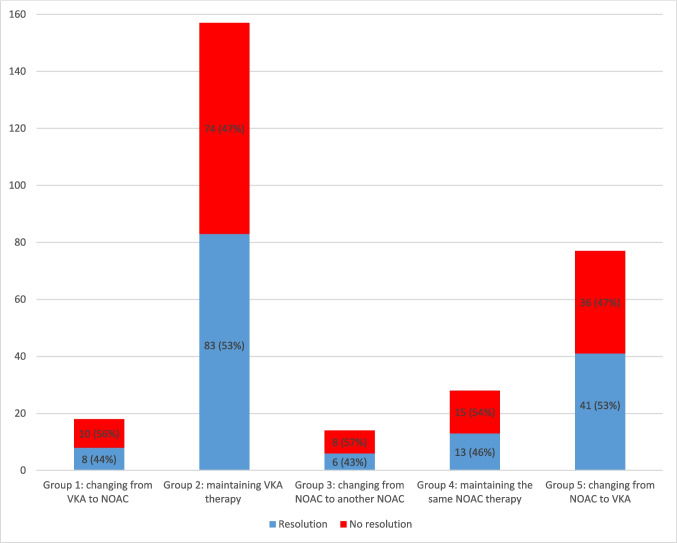
Resolution rate according to treatment change. No statistical relevant difference was observed between the five groups (*p* = 0.866)

**Fig. 2:**
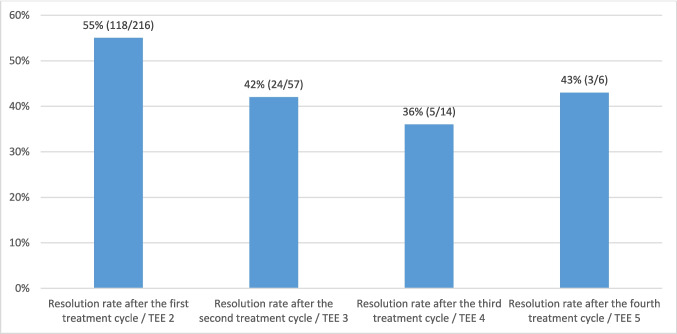
Resolution rate according to treatment cycles. One single patient had five treatment cycles/six TEE examinations with thrombus resolution

Average duration of one treatment cycle was 61.5 days (IQR 50). No statistical relevant difference was observed between the five groups (*p* = 0.866) (Fig. 1). Resolution rate was highest (55%) after the first treatment cycle and declined thereafter (see Fig. 2; *p* = 0.267).

When analyzing cycles with an OAC switch (*n* = 109 (37%)) compared to cycles with no OAC switch (*n* = 185 (63%)), resolution rates were similar (55/109 (50%) vs. 96/185 (52%), *p* = 0.453).

In cases where no thrombus resolution could be achieved (30%, (64/216)), further treatment options were discussed. This included implantation of LAA occluder, which was performed in 14 (6%) patients (implant types: Watchman FLX (Boston Scientific, MA) in five, Amplatzer Amulet (Abbott Medical Inc.) in seven and LAmbre (Lifetech Scientific Corp., Shenzhen, China) in two patients). All patients successfully underwent the procedure, no complications occurred. Rhythm control therapy was successfully pursued in five of these patients. Follow-up TEE showed correct placement with no significant leakage; one patient had device-related thrombosis which resolved after two months of OAC intake. OAC was reduced to single antiplatelet therapy in five cases.

In 13 (6%) other patients, a conscious decision against further rhythm control therapy was made; these patients were given rate-limiting drug therapy and were not scheduled for follow-up TEE. In the rest of the patients (17%, (37/216)), further TEE examinations were scheduled, but patients were lost to follow-up.

### Echocardiographic and laboratory characteristics

Clinical, echocardiographic and laboratory results were compared between patients with thrombus resolution (responders) and patients with thrombus persistence (non-responders, see Tables 1, 2, 3). Overall, left ventricular diastolic dysfunction was common (E/A ratio, mean: 2.46 (SD 1.39)), which was also reflected by left atrial enlargement (left atrial volume index, mean: 48.67 ml/m^2^; (SD 18.13). Furthermore, significant (defined as moderate to severe) tricuspid insufficiency was frequent (31%).

Patients with persistent LAA thrombus exhibited lower TAPSE values (*p* = <0.001), a higher prevalence of significant tricuspid regurgitation (*p* = 0.024), higher CHA₂DS₂-VASc scores (*p* = 0.042), lower LAA flow velocity (*p* = 0.018) and more frequent concomitant amiodarone therapy (*p* = 0.023).

Variables showing statistical significance (TAPSE, tricuspid regurgitation, LAA flow velocity, amiodarone) were considered for the multivariable logistic regression model. However, due to the small number of patients with thrombus persistence who received amiodarone (*n* = 8), this variable was not seen as a robust predictor and was therefore excluded from the multivariate analysis (see Table 4). Additionally, concomitant ASS therapy and CHA₂DS₂-VASc score were included based on clinical relevance ([23], see Tables 2, 3, 4). Multivariate regression analysis identified TAPSE independently predictive of LAA thrombus persistence (OR 0.87; 95% CI 0.78–0.98; *p* = 0.026).

**Table 4:** Uni- and multivariate analysis for left atrial thrombus resolution

Parameter	Univariate OR (95% CI)	*p*-value	Multivariate OR (95% CI)	*p*-value
Female gender	1.16 (0.64; 2.10)	0.622		
Age	1.03 (1.00; 1.06)	0.084		
BMI	0.99 (0.93; 1.05)	0.755		
Paroxysmal AF	0.33 (0.41; 2.73)	0.305		
Coronary heart disease	1.38 (0.77; 2.47)	0.287		
Dilatative cardiomyopathy	1.32 (0.47; 3.73)	0.604		
CHA_2_DS_2_-Vasc score	1.12 (0.97; 1.41)	0.070	1.30 (0.96; 1.77)	0.091
Arterial hypertension	0.86 (0.44; 1.68)	0.654		
Prior TIA/stroke	1.62 (0.73; 3.56)	0.234		
Prior myocardial infarction	1.22 (0.54; 2.78)	0.633		
Prior vein thrombosis	1.20 (0.29; 4.94)	0.804		
Prior pulmonary embolism	1.45 (0.33; 6.24)	0.621		
Diabetes mellitus type II	1.24 (0.66; 2.32)	0.507		
Chronic obstructive pulmonary disease	1.72 (0.77; 3.81)	0.184		
Intake of VKA/NOAC at baseline	1.30 (0.72; 2.34)	0.383		
Switch to any other OAC	1.12 (0.64; 2.01)	0.641		
Concomitant intake of ASS	0.55 (0.28; 1.01)	0.082	0.95 (0.31; 2.90)	0.93
Concomitant intake of clopidogrel	0.64 (0.27; 1.50)	0.304		
Amiodarone	4.17 (1.31; 13.29)	0.016		
eGFR	1.00 (0.99; 1.00)	0.694		
Thrombocyte count	0.97 (0.92; 1.00)	0.120		
NT-pro BNP	1.07 (1.00; 1.14)	0.050		
INR before TEE 1	1.23 (0.69; 2.18)	0.260		
LVEF	0.99 (0.98; 1.02)	0.666		
Moderate to severe aortic valve insufficiency	1.40 (0.40; 4.85)	0.599		
Moderate to severe aortic valve stenosis	1.61 (0.45; 5.73)	0.465		
Moderate to severe mitral valve insufficiency	1.01 (0.58; 1.97)	0.833		
Moderate to severe tricuspid valve insufficiency	2.04 (1.01; 3.92)	0.032	0.86 (0.31; 2.39)	0.77
Interventricular septum diameter	1.12 (0.99; 1.26)	0.070		
Left ventricular end-diastolic diameter	1.01 (0.99; 1.03)	0.178		
Left atrial volume index	1.00 (0.98; 1.02)	0.692		
Left atrial reservoir strain	0.96 (0.90; 1.03)	0.199		
E/A ratio	1.06 (0.46; 2.45)	0.943		
Tricuspid annular plane systolic excursion	0.85 (0.77; 0.93)	<0.001	0.87 (0.78; 0.98)	0.026
Left atrial appendage flow rate	0.552 (0.35; 0.87)	0.014	0.69 (0.38; 1.26)	0.23
LAA ostium diameter	1.05 (0.94; 1.12)	0.386		
LAA length	0.99 (0.94; 1.05)	0.869		
Chicken-wing morphology	2.20 (0.80; 5.69)	0.132		
Cactus/cauliflower morphology	1.33 (0.67; 2.61)	0.410		
Windsock morphology	0.50 (0.24; 1.01)	0.067		
Thrombus located at apex	0.88 (0.44; 1.74)	0.711		
Thrombus located at ostium	0.32 (0.07; 1.50)	0.142		
Thrombus located at body	1.62 (0.81; 3.24)	0.176		
Mobile thrombus	1.55 (0.61; 3.98)	0.358		
Grade I (soft) thrombus	0.95 (0.52; 1.73)	0.856		
Grade II thrombus (<50% of the LAA)	1.05 (0.58; 1.90)	0.883		
Grade III thrombus (>50% of the LAA)	1.19 (0.11; 13.37)	0.888		
Thrombus width	0.99 (0,94; 1.04)	0.675		
Thrombus length	0.99 (0.92; 1.07)	0.841		
Sinus rhythm at TEE1	0.33 (0.04; 2.73)	0.305		

*AF* atrial fibrillation, *BMI* body mass index, *eGFR* estimated glomerular filtration rate, *LVEF* left ventricular ejection fraction, *NOAC* novel oral anticoagulation, *OAC* oral anticoagulation, *TEE* transesophageal echocardiography, *TIA* transient ischemic attack, *VKA* vitamin-K antagonist

### Safety endpoints

After a mean follow-up of 130 (SD 195) days, major bleeding was observed in 3% (6/216) patients: zero in group 1, two in group 2 (BARC 2, 3a), zero in group 3, two in group 4 (BARC 2, 3a) and two in group 5 (both BARC 3a). Four patients required blood transfusion.

Ischemic stroke occurred in one case (0.5%), whereas hemorrhagic stroke was not observed. The ischemic stroke affected the left middle cerebral artery and caused transient symptoms.

## Discussion

To the best of our knowledge, this is the largest analysis comprising anticoagulation strategies in patients with LAA thrombi despite ongoing OAC. After a mean follow up time of 130 (SD 195) days, LAA thrombi resolved in 70% (152/216). Resolution rate after the first treatment cycle was highest at 55% and declined thereafter. However, given the substantial resolution rate even after thrombus persistence was seen in the second TEE, further TEE examination to assess thrombus resolution seems feasible. No significant differences regarding resolution rate between the five different anticoagulation strategies were observed (*p* = 0.866). Most echocardiographic markers, including LAA morphology and thrombus size, had no significant impact on resolution rate, while TAPSE as a surrogate marker for right ventricular function remained an independent predictor of thrombus persistence.

### Previously published studies on patients with persisting left atrial appendage thrombus

Four studies with a design comparable to the present study were identified ([8–10, 24], see Table 5); whereas all other publications were limited to case reports, small case series, or studies including fewer than 40 patients [25–30].

**Table 5:** Comparison of different studies regarding treatment of LAA thrombi

Study (year)	Design	Number of patients under chronic OAC intake	Baseline OAC	Groups	Outcome regarding effectiveness of treatment changes
Nelles (2020)	Retrospective	67/78 (11 patients anticoagulation naïve)	VKA (41%), NOAC (45%)	1: change of OAC; 2: no change	Change of OAC achieved thrombus resolution more frequently (68.4% vs. 42.4%; *p* = 0.04)
Kołakowski (2021)	Retrospective	127/129 (2 patients with heparin)	VKA (42%), NOAC (58%)	1: switch to different OAC or heparin; 2: switch to similar OAC; 3: combination with antiplatelet therapy; 4: no change	Any change (groups 1+2+3) vs. no change (group 4) was more effective (OR 2.97 [95% CI 1.07; 8.25]; *p* = 0.031)
Komlósi (2025)	Retrospective	120	100% NOAC	1: change of OAC; 2: no change	Change of OAC was an independent predictor of thrombus resolution (OR 5.28 [1.55–18], *p* = 0.01)
Gawałko (2026)	Prospective	116 (94 (81%) patients under chronic OAC)	VKA (32%), NOAC (68%)	1: switch from VKA to NOAC; 2: maintaining VKA; 3: switch to heparin	Changing VKA to NOAC led to higher resolution (*p* = 0.022)
*Present study*	Retrospective	216	VKA (53%), NOAC (47%)	1: VKA ➔ NOAC; 2: NOAC ➔ different NOAC; 3: NOAC ➔ VKA; 4: maintaining the same NOAC; 5: maintaining VKA	No significant differences between anticoagulation strategies

*OAC* oral anticoagulation, *NOAC* novel oral anticoagulation, *VKA* vitamin-K antagonist

When diagnosis of LAA thrombus is made despite chronic OAC intake, clinicians have to decide whether to change or to maintain OAC. The majority of previously published studies report that any change in treatment seems to be more effective. However, the anticoagulation regimens and groups used for statistical comparison show great variability, rendering direct comparison difficult.

Nelles et. included 67 patients with chronic OAC intake and reported that OAC change led more often to thrombus resolution than an increase in NOAC dose or target INR (*p* = 0.04). However, the number of patients with OAC change was low (VKA➔NOAC in 10 patients, NOAC➔NOAC in 5 patients and NOAC➔VKA in 4 patients). Importantly, these results were not included in a multivariate analysis, and their independent effect on thrombus resolution in this study remains uncertain.

Similar findings were reported in the so far largest study published by Kołakowski et al. [9] in 2021. Any change in OAC was associated with a higher LAA thrombus resolution rate than no change. Notably, this finding was derived from a pooled analysis (groups 1–3), which was collectively compared with a relatively small group of patients without treatment modification (group 4). The latter comprised 27 patients and was characterized by a low absolute number (*n* = 5) of events (thrombus resolution). Additionally, in 26% of patients with OAC change, a combination therapy with one or more antiplatelet agents was implemented. The same approach was used in 11% in a recently published study by Komlósi et al. [8] who included patients receiving only NOAC therapy. Any modification of therapy (including transition from NOAC to VKA, switching to different NOAC or adding antiplatelet therapy) was an independent predictor of thrombus resolution (OR 5.28 (CI 1.55–18), *p* = 0.01). In our clinical practice, combining OAC with antiplatelet agents is not routinely used due to evidence showing increased bleeding risk [31].

Lastly, a prospective study from 2025 reported favorable results for switching from VKA to NOAC compared to remaining on VKA or switching to low-molecular-weight heparin (*p* = 0.022) [24]. The difference was driven by a high prevalence of LAA thrombus among patients treated with heparin (75%). Usage of continuous subcutaneous anticoagulation appears impractical for long-term management, is not used in our clinical practice and therefore was not included in our study.

In summary, when interpreting our data in the context of previously published studies, no clear or consistent therapeutic strategy emerges. Our results show that LAA thrombus resolution is achieved in a considerable proportion of patients irrespective of the anticoagulation strategy used, suggesting that routine changes in oral anticoagulation may be unnecessary. As a result, current clinical practice remains largely empirical, highlighting the need for prospective, randomized studies to inform evidence-based management.

### Factors predicting thrombus resolution

Whereas several factors (LAA peak flow velocity, advanced age, presence of permanent AF) have been shown to correlate with LAA thrombus resolution in OAC-naïve patients [3, 32], only a few independent predictors in patients with LAA thrombus despite adequate anticoagulation have been identified. Kołakowski et al. reported left atrium area (OR 0.908 [95% CI 0.842–0.979]; *p* = 0.012) and number of treatment cycles (OR 0.457 [95% CI 0.239–0.872]; *p* = 0.017) to be independent predictors for thrombus resolution [9].

In our study, we report for the first time that lower TAPSE is independently associated with thrombus persistence. TAPSE has a positive correlation with left ventricular ejection fraction and an inverse correlation with NT-pro BNP levels [33–35], reflecting more advanced global cardiac dysfunction.

## Conclusion

This is the largest cohort of patients with LAA thrombi despite chronic OAC intake. Thrombus resolution was achieved in 70% and was not significantly affected by OAC regimen. Multivariate regression analysis identified impaired TAPSE to be independently predictive of LAA thrombus persistence.

## Limitations

First, the retrospective study design represents an inherent limitation, particularly as patient adherence to anticoagulation therapy could not be reliably assessed. However, this would represent a random rather than systematic error and is unlikely to have biased our results. In addition, 6% of patients underwent left atrial appendage occlusion (LAAO) despite persistent thrombus, precluding assessment of thrombus resolution. This strategy remains an individual decision without clear guideline-directed recommendations [36]. These patients were counted as cases with thrombus persistence. Clinical characteristics (as assessed in the univariate analysis) were comparable to the rest of the study population.

In our study cohort, half of patients were treated with VKA. Current guidelines recommend the use of NOAC except for patients with mechanical heart valves or mitral stenosis [11] and NOAC are currently prescribed in about 85.5% of cases with a rising prevalence [37]. However, in cases of persistent LAA thrombus despite NOAC therapy, switching to VKA remains the most frequently adopted treatment strategy [5].

Also, due to lack of data in this retrospective setting, we assessed right ventricular function using only TAPSE and could not incorporate strain values or systolic excursion velocity. However, TAPSE is easy to obtain with low inter- and intraobserver variability and correlates with other markers of right ventricular function [38, 39].
